# Skin Cancer Prevention across the G7, Australia and New Zealand: A Review of Legislation and Guidelines

**DOI:** 10.3390/curroncol30070450

**Published:** 2023-06-23

**Authors:** Santina Conte, Ammar Saed Aldien, Sébastien Jetté, Jonathan LeBeau, Sauliha Alli, Elena Netchiporouk, François Lagacé, Philippe Lefrançois, Lisa Iannattone, Ivan V. Litvinov

**Affiliations:** 1 Faculty of Medicine and Health Sciences, McGill University, Montréal, QC H3G 2M1, Canada; santina.conte@mail.mcgill.ca (S.C.);; 2 Faculty of Medicine, University of Toronto, Toronto, ON M5S 1A8, Canada; 3 Division of Dermatology, McGill University Health Centre, Montréal, QC H4A 3J1, Canada

**Keywords:** melanoma, skin cancer, sun protection, legislation, guidelines, G7, taxation, shade provisions, children tanning salons, bans, work safety, ultraviolet radiation, sunscreens, rashguard, work safety, occupational sun exposure

## Abstract

Incidence rates of melanoma and keratinocyte skin cancers have been on the rise globally in recent decades. While there has been a select focus on personal sun protection awareness, to our knowledge, there is a paucity of legislation in place to help support citizens’ efforts to protect themselves from the harmful effects of ultraviolet radiation (UVR). Given this, we conducted a comprehensive review of legislation and guidelines pertaining to a variety of sun protection-related topics in countries of the Group of Seven (G7), Australia and New Zealand. Australia was the only country to have banned tanning beds for individuals of all ages, while other select countries have instituted bans for minors. In workplace policy, there is very little recognition of the danger of occupational UVR exposure in outdoor workers, and thus very few protective measures are in place. With regard to sports and recreation, certain dermatological/professional associations have put forward recommendations, but no legislation was brought forward by government bodies outside of Australia and New Zealand. With regard to youth, while there are various guidelines and frameworks in place across several countries, adherence remains difficult in the absence of concrete legislation and standardization of procedures. Finally, only Australia and a few select jurisdictions in the United States have implemented sales tax exemptions for sunscreen products. In light of our findings, we have made several recommendations, which we anticipate will help reduce the rates of melanoma and keratinocyte cancers in years to come. However, minimizing UVR exposure is not without risk, and we, therefore, suggest the promotion of vitamin D supplementation in conjunction with sun protective practices to limit potential harm.

## 1. Introduction

In recent decades, several countries have noted increasing incidence rates of melanoma and non-melanoma skin cancers while also recognizing that the majority of these derive from extensive exposure to UVR [1,2,3,4,5,6,7,8,9,10]. In a 2020 study conducted by the Global Cancer Observatory of the International Agency for Research on Cancer, the following age-standardized incidence rates for both melanoma and non-melanoma skin cancer were noted amongst countries of the Group of Seven (G7), which comprises seven of the world’s most advanced economies (Canada, France, Germany, Italy, Japan, the United Kingdom and the United States), as well as New Zealand and Australia, in cases per 100,000 individuals: Canada 72.8, France (metropolitan) 36.9, Germany 51.8, Italy 26.4, Japan 2.7, UK 39.8, USA 81.5, Australia 176.6, and New Zealand 159.1 [11]. In the 1980s, Australian councils became increasingly aware of the threat of skin cancer in their communities and created the Slip! Slop! Slap! and SunSmart programs to combat the country’s increasing skin cancer incidence rates [12]. In recent years, studies have found that rates of invasive melanoma are declining in younger Australians [13,14]. Prior to the initiation of the Slip! Slop! Slap! and SunSmart campaigns, the age-standardized incidence rate (ASIR) of melanoma was calculated to be 27 cases per 100,000 in 1982, increasing to 49 cases per 100,000 in 2016 [15]. However, while the age-standardized incidence rate of melanoma, which was standardized to the World Health Organization (WHO) 2000–2025 population standard, has increased across the country, it has decreased for individuals under the age of 40, demonstrating the positive impact that these campaigns have had on younger Australians [15]. A second study, which analyzed population-based incidence trends of keratinocyte cancers in Australia, found that while rates have increased by 2–6% per year over the last three decades, most cases are attributable to individuals over the age of 55 who did not grow up with the Slip! Slop! Slap! and SunSmart campaigns [16].

For many decades, laws have served as critical interventional tools to attain public health goals [17]. Specifically, laws and regulations can educate the general public, deter, generate incentives, promote safer product production and usage, and change the environment, may it be economical or informational [18]. It is claimed that every public health achievement of the twentieth century by the United States Centers for Disease Control and Prevention (CDC) was partially due to legal interventions [19]. That said, Australia has been at the forefront of implementing regulatory framework on sun protection in multiple domains, including banning commercial solariums, implementing favorable taxation policies to promote the sales of sunscreen, regulating sunscreen production to ensure their effectiveness, shade development and elimination or minimization of UVR exposure in the workplace, early childhood services and schools, as well as sports and recreation. As with any other interventional tool, laws and regulations can be implemented at various levels, as in Australia. Specifically, at the individual level, laws can be used to influence behavior through education, incentives, and punishment. At a greater level, they can be used to alter the economic, physical, social, and informational environment [18].

This article reviews the legislation in place at various levels regarding sun protection across countries of the international G7, New Zealand and Australia, a world leader in sun protection initiatives. We conducted a comprehensive review of a variety of legislations across the G7, Australia and New Zealand and compared our findings for these countries below. The selected legislations highlight the vast means and levels at which laws and regulations are implemented in countries with advanced economies, showcasing the flexibility and variability of laws as an interventional public health promotion tool.

## 2. Methods

We conducted a comprehensive review of sun protection legislation and guidelines in the aforementioned countries using a search engine and legal databases. Specifically, as previously described by Diehl et al., we initially used Google as a search engine to find relevant guidelines and then legislations on official governmental websites [20]. For each country and legislation/guideline concerning the ban of commercial solaria, workplace and recreational UVR exposure, UVR-protective wear, sunscreen, UVI reporting, sun protection in youth, shade development, and taxation, a separate search was conducted using the same search strategy for each; only the country was amended in each search. For instance, (“Solaria” OR “Tanning Bed”) AND (“Legislation” OR “Law”) AND “[COUNTRY]”, was used as a search strategy for commercial solaria legislation, while (“Solaria” OR “Tanning Bed”) AND “Guidelines” AND “[COUNTRY]”, was used for the guidelines. As for workplace and recreational UVR exposure, the following search strategy was used for legislation: (“Workplace” OR “Recreational”(AND (“Sun” OR “UVR”) AND “Exposure” AND (“Legislation” OR “Law”) AND “[COUNTRY]”, while the following was used for guidelines: (Workplace” OR “Recreational”) AND (“Sun” OR “UVR”) AND “Exposure” AND “Guidelines” AND “[COUNTRY]”. The same was done for sun protection in youth, sunscreen, UVR-protective wear, shade development, and taxation legislation and guidelines.

When unable to access legislation through official governmental sites, using a Google search engine, or if the search yielded no findings, we used the “Legislation” tab in LexisNexis (https://www.lexisnexis.com/en-us/home.page, accessed on 5 April 2023), a legal database, as in Hartsfield et al. [21], to access legislations in each state in the United States, each province in Canada, the United Kingdom, Australia and New Zealand. Country-specific LexisNexis sites were used for each of the five countries. As for France, Germany, and Italy, EUR-Lex, the official European Union’s law database, was used (https://eur-lex.europa.eu/homepage.html, accessed on 5 April 2023). Finally, the Japanese Law Translation Database System from the Japanese Ministry of Justice was accessed for Japan (https://www8.cao.go.jp/pfi/en/link/link_index_e_01.html) (accessed on 29 March 2023).

## 3. Commercial Solariums/Tanning Beds

Since 2009, the World Health Organization has classified indoor tanning beds as class I carcinogens, which are elements known to cause cancer in humans [22]. Moreover, the International Agency for Research on Cancer (IARC) has previously found a 75% increase in the risk of melanoma with indoor tanning beginning during adolescence or early adulthood [23].

In Australia, commercial solaria have been banned in all jurisdictions since January 2015, apart from Western Australia, where the ban came a year later (in 2016). This ban makes it illegal for any person to provide the use of a tanning bed for a fee. The commercial ban, however, does not affect personal ownership or use of solaria. The Cancer Council advises against the use of these solaria, but there is currently no “national call” by local cancer councils for a ban on the private ownership and personal use of solaria [24]. New Zealand and countries within the G7 have not implemented such a ban (Table 1). However, many have passed legislation that prohibits minors from using tanning beds. In Canada, such a ban was individually implemented by each of the provinces/territories, except for Nunavut and Yukon, while Health Canada recommends against the use of tanning beds [25]. Quebec, Ontario, Manitoba, Saskatchewan, Alberta, British Columbia and Prince Edward Island prohibit sunbed use by minors under the age of 18, while Newfoundland and Labrador, Nova Scotia, New Brunswick and the Northwest Territories prohibit their use for those under 19 years old, the age of majority. These legislations were implemented between 2011 and 2018, with Alberta being the last province to enact such legislation. In the United States, 32 states have imposed a strict ban for minors (ages 14–18), while 8 states require parental consent [20].

Moreover, the federal government took action to combat the use of tanning beds under the Affordable Care Act of 2010, whereby amounts paid for tanning services are subject to a 10% tax [26]. Within European G7 members, Italy, France, Germany and the United Kingdom have also banned sunbed use for minors under the age of 18. Italy also banned the use of tanning beds for pregnant women, people with skin cancer or a history thereof, as well as individuals who do not tan or who burn easily from sun exposure [27]. As of 2017, in New Zealand, commercial operators are banned from allowing minors under the age of 18 to use sunbeds. Individuals and operators in violation can face up to the New Zealand dollar (NZD) 2000 and NZD 10,000 in fines, respectively [28]. Unfortunately, no information could be found regarding legislation pertaining to tanning beds in Japan. Tanning salons operate across the country. Many sports gyms are noted to have a tanning bed or booth available. However, tanning is not culturally accepted in Japan, where pale skin is widely accepted as a standard of health and beauty. A notable exception to this trend was a *Ganguro* (Japanese: ガングロ), a fashion trend among young Japanese women that was prominent in the mid-1990s, where a dark tan and contrasting make-up were used [29].

A cost-effectiveness analysis recently conducted across England found that a ban on commercial indoor tanning combined with a public information campaign would result in 1206 fewer cases of melanoma, 207 fewer melanoma-associated deaths and 3987 fewer keratinocyte skin cancers over the lifetime of all 18-year-olds living in England at that time [30]. Additionally, a microsimulation model of individuals aged 14–17 in the United States compared outcomes of banning or not banning sunbed use for minors, which found that full adherence to the ban would prevent over 15,000 melanoma cases and 3300 recurrences and lead to overall savings upwards of USD 205.4 million, taking into account the cost of implementing such a program and the financial losses to the tanning bed industry [31]. Finally, several studies have analyzed the consequences of banning commercial solaria in Australia in the last decade. A study conducted by Gordon et al. found that a ban will help avoid over 31,000 cases of melanoma, and almost 470,000 cases of keratinocyte carcinomas, realize over USD 47 million in savings to the healthcare system and have productivity gains of USD 375 million for young Australians over the course of their lives [32].

## 4. Occupational Health

All Australian states and territories, except Victoria and Western Australia, have enacted local Work Health and Safety Acts (WHS Acts) and Work Health and Safety Regulations (WHS Regulations) based on models developed by Safe Work Australia, an Australian government statutory agency (Table 1). These WHS Acts and Regulations require that persons conducting a business implement policy to eliminate or minimize risks arising from the work environment, including UVR exposure. In turn, employees also have a duty to attend to their own health and safety and cooperate fully with their employers’ efforts. In this respect, employees must follow workplace sun protection policies and practices, attend training, and use supplied protective equipment as instructed. In addition, at the national level, the Australian Radiation Protection and Nuclear Safety Agency (ARPANSA) published radiation protection standards that also require employers to implement sun protection plans, as well as set limits for occupational UVR exposure. According to a report from the Cancer Council, 1360 workers’ compensation claims for sun-related damages were made in Australia between 2000 and 2009, at a total cost of AUD 38.4 million. Many court decisions in these cases are publicly available and firmly establish the legal recognition of sun exposure as an important occupational hazard.

In New Zealand, the Health and Safety at Work Act 2015 (HSWA), largely influenced by Australian work and safety laws, aims to give workers the highest level of protection while at the workplace. Employers are required to provide a risk-free environment, maintain safe structures and systems, and provide information, training, and supervision required to prevent injury or illness. Finally, employers are expected to monitor work and health conditions to prevent any injury or illness at work. Per the HSWA, UVR and heat are considered hazards that could injure workers and make them ill. As such, employers must ensure that their workers are adequately protected while under sun exposure. They should give their workers adequate clothing, sunscreen, protective hat and eyewear, and water [33]. Those in violation of the HSWA could face improvement notices, prohibition notices, prosecution, and fines up to NZD 1.5 million [34].

Across the G7, the regulations pertaining to UVR exposure are less pronounced. According to the Canada Occupational Health and Safety Regulations, there are limited requirements for heat stress and non-ionizing radiation. While employees have the right to refuse dangerous work, such work is defined as having an imminent or serious threat, which would only apply to sun safety in extreme circumstances. It also states that handling equipment that is used outdoors should be fitted with a structure to protect the operator from weather conditions that may be hazardous to the operator’s health or safety but does not specify the nature of the conditions that may be hazardous. Across the Canadian provinces/territories, there are no clear regulations in place that recognize UVR exposure and its link to skin cancer in the workplace. In Newfoundland and Labrador, diseases caused by UVR and occupational cancer are listed as notifiable occupational diseases, but there is no note of the employer’s or employee’s role in reducing or acknowledging such exposure. In the Nova Scotia Health and Safety Act, there are broad duties to provide protection if there is the possibility of injury to the eyes, face, neck or skin, which may expand to sun exposure. In Saskatchewan, when there is a risk of irritation or injury to the face, eyes or skin from UVR or infrared radiation, the employer is to provide industrial eye or face protectors, as well as protective clothing/covers or any other safeguard that provides equivalent protection for the worker. Manitoba also enforces that employers must provide personal protective equipment to employees if there is a risk of injury due to UV or other forms of radiation. In France, Italy, Germany, the United Kingdom and Japan, only ionizing or artificial radiation are considered to be physical hazards and thus discussed by Occupational Safety and Health. Finally, in the United States and as of 2021, there is no Occupational Safety and Health Administration (OSHA) standard regarding exposure to UVR. However, the OSHA provides employers with general duty clauses, one of which states that the workplace must be free from recognized hazards that may cause serious physical harm.

Protecting outdoor workers against UVR has been thought to be crucial in decreasing the incidence rates of both melanoma and keratinocyte skin cancers. Multiple studies have demonstrated the association between occupational exposure and skin cancer, whereby outdoor workers are exposed to UVR doses that are five times above the internationally recommended limits [35]. This has an important impact on health outcomes, whereby outdoor workers have a 77% higher risk of squamous cell dysplasia (actinic keratosis and squamous cell carcinoma) and a 43% higher risk of basal cell carcinoma (BCC) [36,37]. Finally, in 2007 the International Commission on Non-Ionizing Radiation Protection, in collaboration with the International Labor Organization and the World Health Organization, suggested generic protective measures for outdoor workers, ranging from avoiding exposure to direct sunlight to sunglasses with side panels, after realizing that outdoor workers are at increased risk of adverse consequences given their significant exposure to solar UVR [38].

However, select studies have suggested that cutaneous melanoma incidence rates are lower among outdoor workers, as noted in some Nordic countries where individuals with outdoor work had lower standardized incidence ratios than those who worked indoors [39]. Factors found to contribute to higher melanoma incidence rates were high socioeconomic status, as well as technical, transport, military, and public safety workers with potential skin exposure to carcinogens [39]. In addition, one systematic review did not report differences between outdoor versus indoor workers with regard to cutaneous melanoma development [40]. Depending on the country and its geographic latitude, it is likely that a combination of factors (UV index, Fitzpatrick skin phototype, occupational behavior, hobbies, vacation patterns, etc.) impact the overall risk for the development of melanoma, where in some countries (e.g., Nordic countries) the risk of occupational exposure may be lower compared to other countries that are located closer to the equator.

## 5. Ultraviolet Radiation-Protective Textiles Standards

Using textiles as a means of protection from UVR is effective in preventing skin cancers, premature aging, and photosensitive/photoexacerbated disorders. However, despite claiming to be protective, many textiles are deemed inadequate. As such, regulations were needed to ensure that textiles were as protective as they claimed to be [41]. In 1996, Australia and New Zealand set a global labeling standard through the Australian and New Zealand Standard, AS/NZS 4399:1996, by establishing the Ultraviolet Protection Factor (UPF) as a standard measuring the amount of UVR passing through fabric [42] (Table 1). Albeit not mandatory for all, manufacturers selling products as sun protective, with the UPF label, must abide by them. The standard was criticized for not testing garment stretch, lifetime, the effects of water, and taking into account body coverage [43]. As a result, in 2017, the standards were updated to simplify the UPF classification as a minimum, good and excellent. Moreover, minimum requirements for gloves, hats and accessories were added, alongside a minimum requirement for body coverage [44]. In 2020, Australia revised the standard to make its own—without New Zealand. Specifically, the standard no longer labeled women’s one-piece swimsuits as sun protective unless they complied with the body coverage requirements and provided the minimum brim requirements for hats [45].

In 1998, a working group within the European Union (EU) convened to set standards for UVR protective textiles. It generated a standard comprised of two parts; the first covered the tests for textile materials, while the second concerned the classification and labeling of products. Per the standards, a textile product needed to have a UPF greater than 40 and UVR transmission lower than 5% in order to be considered UVR-protective [46]. In 2016, the EU introduced a regulation for personal protective equipment (PPE), creating a debate as to whether UVR protective textiles were included. This was clarified by the working group in 2022, which indicated that personal equipment for UVR protection, as opposed to equipment intended for protection from extreme UVR, is not considered PPE [47]. Similarly, the United Kingdom has two standards in place; one for testing requirements and the other for labeling. Per the standards, only textiles with a UPF of at least 40 can be labeled as UVR-protective [48].

In the United States, the American Society for Testing and Materials (ASTM), assisted by the Federal Trade Commission and the Consumer Products Safety Commission, released three standards concerning UVR-protective textiles. These standards address the manufacturing of such products, their testing, and labeling based on the Australian and New Zealand UPF classification [49]. A product must abide by them in order to be labeled as UVR-protective but cannot, under any circumstances, claim to protect from skin cancer. In Canada, the government’s Centre of the Competition Bureau released the Consumer Guidance on UV Protective textiles. It provides that textiles claiming to be UVR-protective should have a UPF rating, with 15 being the lowest. Moreover, it provides a list of the corresponding percentage of UVR blocked by each UPF rating [50]. Finally, in 2019 the Japanese Standards Association published standards to test and provide a UPF label for UVR-protective textile products [51].

## 6. Sunscreen Regulation

In Australia, sunscreens labeled as therapeutic goods cannot be sold prior to being listed or registered in the Australian Register of Therapeutic Goods (Table 1). Therapeutic sunscreens are primary sunscreens for UVR protection with a Sun Protection Factor (SPF) of 4 or higher [52]. The sunscreens must abide by the Australian/New Zealand Sunscreen Standard, which sets forth labeling and testing requirements. Sunscreens’ active ingredients and composition must be found effective and safe before they are sold [53]. As of September 2022, all manufacturers and importers of sunscreen in New Zealand, just like in Australia, must abide by the Australian/New Zealand Sunscreen Standard [54].

In December 2022, the U.S. Food and Drug Administration (FDA) imposed more regulations on over-the-counter sunscreens through a posted final order. Per the order, sunscreens containing 16 specified active ingredients are deemed safe and effective. The order does not require broad-spectrum testing but grants manufacturers the option to conduct broad-spectrum testing to add the label on products [55]. In November 2022, Health Canada updated its Primary Sunscreen Monograph, as they are considered therapeutic products. The document indicates the requirements necessary to receive authorization to sell primary sunscreen. It provides the ingredients that could be present and the required labeling, such as SPF, cautions and warnings. Moreover, it discusses optional labeling, such as broad spectrum and water resistance and notes that sunscreens with SPFs >50 are to be declared as SPF 50+ [56]. Additionally, it notes that sunscreens with SPFs less than 15 only help prevent sunburn and not skin cancer or early skin aging and that this must be indicated on the packaging with a specific verbatim statement [56].

Given that Germany, France, and Italy are members of the EU, sunscreen products sold therein must abide by the EU Commission Recommendation of September 2006. Specifically, they are considered cosmetic products and must have an SPF of at least 6 and protect from all dangerous UVR. Moreover, products must be labeled with an efficacy classified as low, medium, high, or very high, cautions and warnings [57]. The United Kingdom’s regulations are very closely aligned [58]. Finally, in Japan, although sunscreen products are regulated by the Pharmaceutical Affairs Law, the Japan Cosmetic Industry Association’s non-legally bindings standards are followed by most manufacturers in Japan [59]. They address the means to measure SPF, UVA grade protection, and water resistance [60].

## 7. Taxation Policies

Although sunscreen is vital in skin cancer prevention, its use remains limited. This could be attributed to many factors, one of which is the cost barrier [61]. Patients may require financial assistance to use sunscreen regularly in line with sun protection guidelines [62]. The administration of favorable tax policies could be a means to address that. For instance, in Australia in 2001, sales tax was removed from sunscreens, and in 2002, tax deductions on items, including protective sunglasses pertaining to sun protection for outdoor workers, were implemented [63] (Table 1). On the other hand, while a sales tax is applied to sunscreen products in New Zealand, employers can claim tax deductions for outdoor sun protection items [64], but employees cannot. As for Canada, no province or territory has legislation exempting personal sun protection products from sales tax. In fact, British Columbia’s provincial sales tax exemption regulation specifically states that sunscreen products are not exempt from sales tax. Moreover, Manitoba’s Tax Publication issued a public bulletin listing baby supplies that are not taxable while indicating that sunscreen items are taxed [65].

Six states within the United States have sales tax exemptions for sunscreen products. Specifically, in California, the sale of sunscreen by a dermatologist to a patient is exempt from sales tax, but those purchased over the counter are not [66]. In New York, Virginia and Texas, non-prescription sunscreen is not taxable [67,68,69]. In addition, Maryland provides a sales tax exemption on sunscreen lotion with SPF 15 or higher as it is considered a disposable medical product [70]. Finally, in Florida, consumers can receive tax exemptions on the first USD 15 of the sales price of sunscreen during Freedom Week’s sales tax holiday in the first week of July [71]. On the other hand, similar to Manitoba and British Columbia in Canada, a few states, such as New Jersey and Vermont, explicitly clarify, in official documents, that sunscreen products are not exempt from sales tax [72,73]. As for the other G7 countries, France, Italy, Germany and Japan do not have sales tax exemptions for sunscreen products. In the United Kingdom, the Financial Secretary stated in February 2023 that sales tax exemptions would not be made for sunscreen as they are available through doctors’ prescriptions with no taxes for those in medical need [74].

While removing or adding sales taxes is one of the most common policy tools used to promote healthy behaviors worldwide, as indicated by World Health Organization documents, their use pertaining to sun protection products is limited in G7 countries [75,76]. This is despite several studies highlighting the effectiveness of health promotion via taxation policies. Specifically, measures to increase tax rates on sugar-sweetened products have been found to likely promote healthy behaviors and reduce demand [77]. On the other hand, providing fresh fruits and vegetables to postpartum women at a reduced price increased their consumption over 6 months. While tax measures are effective in promoting healthier behaviors, underlying socioeconomic factors must also be addressed, as health behavior is influenced by one’s environment [78].

## 8. Early Childhood Services and Schools

All Australian states and territories have enacted comparable versions of the Education and Care Services National Law Act 2010 and the Education and Care Services National Regulations (Table 1). Under these, approved providers of education and care services must put in place and follow policies and procedures in relation to sun protection. Additionally, they must ensure that outdoor spaces at the premises include adequate shaded areas to protect children from overexposure to UVR. Guidance on how to develop and implement such policies is found in normative documents adopted by the Australian Children’s Education & Care Quality Authority in the Sun Protection Policy Guidelines and the Guide to the National Quality Framework, Operational Requirements, Quality Area 3 (Physical Environment), 3.6 (Shade). These policies aim to create safe sun environments and align with the Cancer Council’s SunSmart program. In fact, early childhood education and care services, as well as primary schools, are invited to apply for the SunSmart status upon fulfilling certain conditions (e.g., having a written sun protection policy meeting minimum standards, rescheduling/minimizing outdoor activities in direct sun during peak UVR periods of the year, teaching, modeling and reinforcing positive sun protection behavior, and agreeing to undergo regular audits).

While New Zealand has no specific obligations to protect school children from the sun, there are recommendations and guidelines in place [79]. While not explicitly mentioning protection from UVR, the Education (Early Childhood Services) regulation requires licensed school providers to take all reasonable steps and precautions to promote the good health and safety of children [80]. On the other hand, although it’s non-binding, the Ministry of Education informs school boards to install sunshades to protect children [81]. Despite this, it has been found that school children are not adequately protected, and as such, schools are encouraged to take measures such as mandating hats, adopting sun-protective uniforms, and building shades [82]. Although there have been some improvements, sun protection in primary schools is still inadequate, and prevention is not publicly funded [83].

Amongst countries of the G7, most have also created guidelines to create supportive environments for youth. In Canada, all provinces/territories except for Prince Edward Island, Alberta and Yukon have implemented variations of UVR guidelines mandated by legislation for children and childcare workers within the licensed childcare establishments. These include protective clothing for outdoor play, application of sunscreen for outdoor play, modeling of sun protective behaviors by staff, provision of shade in outdoor play spaces, and scheduling of recess based on sunshine and UV index. However, the degree of policy adoption is thought to be low across the country. In Germany, the “UV protection: Clever in sun and shade” project was implemented to share knowledge and practical everyday tips for sun and skin protection in children and youth, whether in daycare centers, schools or sports centers. In the United Kingdom, the *Event Safety Guide* asks organizers to consider whether there is shade and shelter at open-air sites in dedicated children’s areas. Moreover, the *Sun Safe Schools* national accreditation scheme was implemented in the UK in 2013, which aimed to educate children on the importance of sun safety and assist primary schools in implementing a suitable sun-safe policy. It consists of a four-step action plan that schools must complete, whereby upon completion, they are awarded certification with a one-year validity with a possibility of renewal. Furthermore, in 2020, the UK introduced the Personal, Social, Health and Economic Education curriculum, under which all primary schools must educate students about safe and unsafe exposure to the sun and how to reduce the risk of sun damage, including skin cancer. In the United States, the Division of Cancer Prevention and Control from the National Centre for Chronic Disease Prevention and Health Promotion strategized shade planning for America’s schools and provided recommendations (Shade Planning for America’s Schools). No information could be found regarding sun protective practices directed toward youth in France, Italy or Japan.

A randomized trial regarding the effectiveness of the “Clever in the Sun and Shade for Preschools’ Program” (CLEVER) in Germany found significantly stronger rates of sun protection behaviors in preschools taking part in the program, fewer perceived impediments to avoid the sun, and higher self-efficacy to use sunscreen [84]. Furthermore, a review of the literature on interventions towards sun-protective behaviors in American youth found significant increases in positive behavioral changes (i.e., increases in sunscreen application, use of hats and sun-protective clothing, shade-seeking, avoidance of outdoor activities during peak UV radiation period), increased knowledge, changes in attitudes towards tanning, and decreased sun exposure repercussions (i.e., new sunburns, number of new nevi, change in pigmentation of the skin) [85], demonstrating how pivotal it is to educate youth about the importance of sun protection.

## 9. Ultraviolet Radiation Index Monitoring

The ultraviolet radiation index (UVI), developed in Canada, is an internationally adopted measure of UVR [86] (Table 1). It has been widely available to the public to assist in making informed sun-protective decisions [87]. Knowledge of UVR levels can be vital in promoting sun-protective behavior [88]. In Australia, UVI and protection times are provided in weather forecasts by the Bureau of Meteorology. Members of the public are recommended to seek sun protection when the UVI is above 3. One can also find detailed 3-h UV Index forecasts [89]. New Zealand’s NIWA Weather, a public weather forecasting, provides UVI for the day and forecasts. The levels are provided in a graph for each hour [90]. In Canada, Environment and Climate Change Canada publishes a daily UVI forecast for major cities and towns. If the UVI is greater than 1, it is published on Environment Canada’s weather forecast and included on television, radio, and newspaper forecasts [91]. In the United States, The National Weather Service calculates the UVI forecast, and the United States Environmental Protection Agency publishes it online. Daily and hourly UV Index values are provided for more than 50 cities [92].

In Europe, more than 160 stations in 25 European countries monitor the UV Index, with more than 57% of the population having access to the data online. In the United Kingdom, monitoring is carried out by the University of Manchester in 14 stations on behalf of the Department of Environment Food and Rural Affairs. In Germany, UV monitoring is carried out by the Federal Office for Radiation Protection, German Environment Agency, and the German Weather Service network. In Italy, UVI monitoring and publishing are carried out by more than five institutions, such as Agenzia Regionale per la Protezione Ambientale and the Atmospheric Sciences and Climate from the National Research Council. Updates are published online every 5, 10, 15, or 30 min, depending on the monitoring institution. In France, UVI monitoring is carried out by three stations. As the measurements are not adequate, UVI is provided to the public as a forecast instead of measurements [93]. In Japan, the UV Index forecast, Clear Sky UV Index Forecast, and UV Index Estimate are collected and displayed on an interactive map online by the Japan Meteorological Agency [94].

## 10. Sports and Recreation

In Australia, event organizers are considered as persons conducting a business or an undertaking under the WHS Acts and Regulations and have a similar duty of care as employers (Table 1). According to SunSmart, such responsibility may involve providing participants, patrons, staff, and volunteers with protection from overexposure to UV radiation. Despite decades of sun protection campaigns in Australia, one study found the use of sun protection in most outdoor sports is inadequate [95]. A few Australian organizations have been providing sunscreen via dispensers, for free or at a discounted price, to the public. For instance, the Can Too Foundation partnered with Sunscreen Stations Australia to install automatic sunscreen stations at several beaches, schools, and parks [96]. Such initiatives ensure that sunscreen protection is available and remain affordable.

Similarly, in New Zealand, event organizers could be considered as a “person conducting a business or undertaking”, and as such, have the same obligations towards members of the public as they do towards their employees [34]. Despite that, children’s playground equipment, sand areas, and pools were found to have very few shaded areas [97]. Similar to Australia, with regard to publicly available sunscreen, SkinCan New Zealand installed several dispensers across Christchurch [98].

Across the G7, no legislation was found with regard to sun protection in the realm of sports and recreation. In Canada, the Canadian Dermatology Association (CDA) recommends that sporting activities or training be scheduled outside of 11 AM to 4 PM when the sun’s rays are the strongest. However, the CDA mostly makes recommendations targeted to participants, suggesting that shade be sought whenever possible, that sun-protective clothing be worn and that sunscreen and lip balm with an SPF of 30 or higher be applied. In 2022, the Save Your Skin Foundation launched a sunscreen dispenser pilot program. The ten touchless and automatic dispensers were installed in British Columbia, Prince Edward Island, Alberta, and New Brunswick to provide free sunscreen to the public [99]. Finally, in Ontario (Toronto), through the #BeSunSafe project, multiple sunscreen dispensers were installed in popular public spaces.

The American College of Sports Medicine (ACSM) makes similar recommendations to the CDA. In the US, certain states and cities, such as Los Angeles, offer heavily-discounted or free parking at beaches after 4 PM [100]. Moreover, several cities, such as Miami, New York, and Boston, have installed free sunscreen dispensers in public places [101]. It has been reported that IMPACT Melanoma, a non-profit organization, assisted in the installation of more than 1500 dispensers across the United States, highlighting an increasing trend [102].

Several studies have drawn associations between sports and recreation and an increased risk of skin cancers. A study conducted on a Southern European population found that beach holidays and winter sports were independent risk factors for BCC [103]. Likewise, a study conducted in Switzerland found that sun exposure during outdoor sports showed a twofold increased risk of BCC [104]. Moreover, other studies have found that a history of winter sports in childhood carried an increased risk of skin cancers [105], while participation in outdoor sports was identified as an independent risk factor for melanoma [106], reinforcing how important sun protection is in the world of sports and recreation.

## 11. Public Shade Development

Local governments in Australian states and territories are encouraged to take action to reduce community exposure to UVR (Table 1). In Western Australia, the local Cancer Council adopted guides that list policies that local governments may implement, which include a comprehensive shade policy that covers all local government-owned or managed buildings and facilities, a sun protection policy for all local government-sponsored, funded or organized community events, and a workplace policy for staff, volunteers and elected officials who participate in outdoor work or activities. Moreover, the SUNbusters initiative, funded by Queensland Health, provided seed grants of AUD 500 to community and sporting not-for-profit organizations to build shade for children [107]. In addition, the Queensland Cancer Council and the Queensland Government supported the SunSmart Shade Creation Initiative AUD 25,000 permanent shade grant for not-for-profit organizations working with minors [108]. The Victorian government has also launched a shade grant program for schools and community shade. Since 2015, the program awarded more than 2300 grants worth AUD 20 million [109]. Finally, the Queensland Government Department of Health published a series of technical guidelines for shade provision in public facilities, which describes the appropriate shade type and location per type of public facility [110].

In New Zealand, there are no governmental guidelines or legislation concerning public shade. However, the Ministry of Education provides that schools must seek community grants while not specifying the means to help install shade structures [81]. The Cancer Society requested that government-funded schools install shades and provide them in public spaces, including at events [111]. Consistent with this aforementioned need, playgrounds in New Zealand’s capital city have been deemed to have insufficient shade [112].

In Canada, eleven municipalities have at least one policy that incorporates the provision of natural (trees) and/or artificial shade structures in land use planning and public facilities (parks, streets). Only two municipalities, namely Toronto and Halifax, indicate skin cancer prevention as a benefit of these initiatives. In 2020, the Canadian Dermatology Association distributed a total of CAD 41,200 in grants to build and install permanent shade structures in outdoor areas. In the United States, the American Academy of Dermatology runs a similar grant program to that seen in Australia, whereby a CAD 8000 grant is provided to each successful applicant for shade provision projects [113]. No legislation, guidelines or initiatives pertaining to shade in the remaining countries of the G7 were found.

The importance of shade in the prevention of skin cancer has been well established. In 2014, the United States surgeon general issued the Call to Action to Prevent Skin Cancer, in which three of the five strategic goals addressed the importance of shade [114]. In a study conducted in the United States between 2010 and 2020, American adults had a significantly increased prevalence of seeking shade, alongside other sun-protective behaviors [115]. Although the promotion of shade-seeking is of utmost importance, more action must be taken by local governments to ensure that citizens have access to such spaces.

## 12. Discussion

In light of the aforementioned findings and given the positive influence of laws on the promotion of public health, we suggest the implementation of laws and regulations at various levels to protect the general public, educate them, and raise awareness in the G7 to a similar degree as seen in Australia (Table 2). Our first recommendation is the universal banning of commercial tanning beds for all ages, seeing the positive impact this intervention has had in Australia. Second, in terms of occupational health, employees should be provided with training and education, and laws and regulations should be instilled to minimize, as much as reasonably possible, UVR exposure, whether by limiting time spent outdoors, by not working in peak UV index hours, or by mandating the use of sun protective clothing. Third, concerning sunscreen, sales taxes should be removed to ensure that sunscreen is as affordable as possible. Seeing that sunscreen plays a role in preventing cancer and thus saving money for the healthcare system in the long term, sunscreen should be considered an essential product, and we recommend that prices be better regulated by governing bodies to avoid price gauging. Pricing aside, stringent labeling and testing requirements must be implemented to ensure product efficacy and consumer safety and awareness, and inefficient levels of SPF (e.g., SPFs < 15) should be indicated to avoid a false sense of security in patients. Fourth, public shade areas should continue to be developed in schools, recreational areas and sports spaces. Funding should also be available to individuals and entities seeking to build shaded areas. Lastly, we suggest mandatory labeling of all sun-protective clothing with a UPF label to ensure customer protection and awareness. Overall, we believe that investing in these recommendations at the present time will not only have a positive impact on generations of patients but will save healthcare systems around the world time and resources/money in the future.

However, strict public health legislation may have unintended negative consequences on individuals and businesses. Specifically, it can backfire, pushing individuals away from healthy behavior. A study in 2020 found that public behavior is greatly influenced by political ideology; for example, self-identified conservatives in the United States were more likely to not view governmental legislation in a positive light. For instance, they viewed vaping more favorably following new laws on warning labels. One can therefore argue that the use of less strict legislation may increase compliance. As such, governments may focus on communicating effectiveness as opposed to implementing stricter regulations [116]. As for the impact on businesses, it has been found that regulations may impose a burden, disproportionately affecting small businesses [117]. In this case, manufacturers of sunscreen and sun protective clothing or equipment may be significantly impacted should individuals turn away from such products due to legislation that is too strict. Additionally, studies have found that health and safety regulations have had a negative impact on the United States gross national product, whereby occupational health and safety regulations have previously caused a significant reduction in manufacturing productivity [118].

Since the discovery of UV radiation’s involvement in skin cancer, many primary prevention programs have emphasized the importance of UV avoidance. However, several positive, systemic effects have been noted in the literature, which begs the question: might sun-avoidant behaviors have an effect on an individual’s general health? Among the most important of sunlight exposure’s benefits is the generation of vitamin D, whereby substantial deficiencies can precipitate and exacerbate osteopenia and osteoporosis and increase the risk of fractures, as well as serious consequences such as the increased risk of common cancers, autoimmune diseases, infectious diseases, and cardiovascular disease [119]. Cancers hypothesized to be associated with a lack of vitamin D include Hodgkin lymphoma, as well as breast, ovarian, colon, pancreatic and prostate cancers, whereby living at higher latitudes increases an individual’s risk of dying from these malignancies [120]. A randomized clinical trial published in 2007 found that taking 2–4 times the daily dietary reference intake of 200–600 IU of vitamin D_3_ and calcium resulted in a 50–77% reduction in expected incidence rates of all cancers in a four-year period [120], demonstrating that vitamin D may be obtained from diet/supplements in order to exert a positive impact.

Moreover, recently researchers found that UVR exposure led to increased libido and boosted levels of sex hormones in both men and women, which may have impacted human courting/dating practices [121]. These findings were corroborated in mouse models, whereby UVB exposure increased circulating sex steroid levels, enhanced female attractiveness and receptiveness towards males and increased the female estrus phase, hypothalamic–pituitary–gonadal axis hormones, and follicle growth [122]. Finally, studies have found associations between UVB exposure and depression, whereby UV-B exposure at normal levels improved mood and depression [123,124,125]. However, the authors acknowledge that high levels of UVB exposure can result in numerous diseases, such as skin cancer, which can, in turn, increase the risk of depression [123].

It is UV exposure’s addictive potential that makes it especially dangerous. A rodent study, which was corroborated in humans, found that β-endorphins were synthesized in the skin following exposure to low-dose UV [126], increasing pain-related thresholds. A study conducted in 2005 found that 26% of surveyed beachgoers met modified Cut, Annoyed, Guilty, and Eye (CAGE) criteria for substance abuse in the context of UV-seeking behaviors, demonstrating 2+ hallmarks of addiction. Moreover, 53% met the American Psychiatric Association’s Diagnostic and Statistical Manual of Mental Disorders (DSM) criteria for substance abuse by responding positively to 3+ of 7 signs of addiction [127].

When weighing the risks and benefits associated with UV radiation, we must consider the mortality rates of the various forms of skin cancer in comparison to the mortality associated with the above-mentioned negative impacts of UVR deficiency. While BCCs and SCCs have high incidence rates, their mortality rates are relatively low: the 5-year relative survival for BCC, SCC and melanoma is 100%, 95% and 89%, respectively, according to the Canadian Cancer Society [128,129]. However, when assessing the mortality rates of some of the diseases associated with long-term vitamin D deficiency, they are found to be much more morbid, with 5-year survival rates of 67% for colorectal cancer [130], 89% for breast cancer [131], or 45% for ovarian cancer [132], or require more extensive treatment modalities to be cured, such as large surgical removal, radiation or chemotherapy. Given its anti-inflammatory, immunomodulatory and antiangiogenic effects, some hypothesize that vitamin D may act as a carcinogenesis inhibitor [133].

In sum, in order to decrease skin cancer rates while protecting individuals against the potential harms of vitamin D deficiency, it is important to emphasize the importance of adequate vitamin D supplementation. Although non-melanoma skin cancers carry very low morbidity and mortality, melanoma skin cancers do, and overall such cancers are very costly to the healthcare system, whereby USD 8.9 billion was spent on treating both melanoma and keratinocyte skin cancers in the United States in 2018 alone [134]. By promoting both sun protective practices and vitamin D supplementation, we can protect against skin cancers and a variety of other diseases, conferring the greatest degree of protection.

Additionally, when discussing UV radiation and its relationship with skin cancer, it is important to acknowledge that the different forms of skin cancer are associated with different sun exposure profiles [135]. For instance, a history of sunburns below the age of 25 was associated with an increased risk of all skin cancers, whereas a history of severe sunburns over the age of 25 was associated with an increased risk of basal cell carcinoma (BCC) and squamous cell carcinoma (SCC) primarily [135]. When comparing keratinocyte skin cancers, cumulative lifetime UV exposure is thought to be an important factor in the development of SCC [136], whereas BCCs are more so associated with intermittent episodes of intense UV exposure [137]. Understanding sun exposure profiles is important because it allows for risk stratification and appropriate public policy to be implemented. For instance, male outdoor workers have been found to have an increased risk of SCC [138], reinforcing the importance of shade, sunscreen, and sun-protective clothing. However, for some Nordic countries, a study conducted among Swedish construction workers found that this population had no increased risk of keratinocyte skin cancers, despite the extensive time spent outdoors, as well as no increased risk of melanoma, except for tumors on the head, face and neck in the high exposure group [139]. Also, a study conducted in Denmark found a decreased risk of keratinocyte skin cancers amongst outdoor workers [140], suggesting that for this northern country, outdoor work may not lead to an increased risk of SCC.

The major limitation of our analysis was finding legislation in various countries. Some were readily accessible through legal databases, while others were found on various websites. Additionally, accessing official English translations of laws in France, Germany, Italy, and Japan was challenging. Even when accessed, some context and words may have been lost in translation. It is important to note that beyond legislation, litigation serves as an important tool to enforce and develop laws [141]. However, no litigation challenging enacted laws and regulations were found online. This could be the case if no lawsuits were filed or if such lawsuits did take place but were not published for privacy and confidentiality reasons.

## 13. Conclusions

Skin cancer is universal as it has no respect for class or gender or, to a more limited extent, skin type, and it is exceedingly common for most people to know someone who has had skin cancer. It is preventable with simple and effective steps that people can directly take. There are very few commercial opponents to sun protection. On the contrary, many commercial opportunities have sprung from the increasing demand for shade structures, hats, protective swimwear/sportswear, sunscreen, and sunglasses. The messages of sun protection are not perceived as politically threatening and involve little or no erosion of tax revenue, nor are they likely to diminish voter support or financial contributions. Hence, despite outlined positive advancements in legislative efforts, more should be done to support individuals in their efforts to protect themselves from UVR. Specifically, countries should legislate sun protective behaviors and infrastructure in schools and childcare settings, as well as in the world of sports and recreation. Moreover, stringent sunscreen and UVR-textile manufacturing standards must be enacted for consumer protection. Finally, favorable taxation policies for sun protective equipment and over-the-counter products should be implemented to ensure availability and affordability, on top of advocating to ban sunbeds. However, it is important to realize that vitamin D deficiency, through lack of UVR exposure, is not without harm and that campaigns that aim to have individuals limit their sun exposure time should also promote vitamin D supplementation to counter potential harmful effects. These key efforts will greatly help reduce the rates of melanoma and keratinocyte cancers in years to come.

## Figures and Tables

**Table 1:** A Summary Table of Legislation and Guidelines Across Australia, New Zealand and Countries of the G7.

Country	Commercial Tanning Beds	Occupational Health	Taxation Policies	Sunscreen Regulation	Early Childhood Services and Schools	Sports and Recreation	Public Shade Development	UVR-Protective Textiles	UVI Monitoring
Australia	Banned in all jurisdictions since 2016	Work Health and Safety Acts require policies that eliminate/minimize risks arising from the work environment, including UVR exposure, enact sun protection policies and practices, provide training sessions, supply protective equipment ARPANSA requires employers to implement sun protection plans, set limits for UVR exposure	Sales tax removed from sunscreen in 2001 Tax deductions provided for protective clothing for outdoor workers in 2002	Sunscreens must abide by the Australian/New Zealand Sunscreen Standard’s labeling and testing requirements Ingredients and composition must be effective and safe before they are sold	Education and care service providers must put in place and follow policies and procedures related to sun protection; outdoor spaces must have adequate shaded areas to protect children from overexposure to UVR Services and schools are encouraged to apply for SunSmart status	Under the Work Health and Safety Act, similar duty of care as employers SunSmart states that such responsibility may involve providing participants, patrons, staff and volunteers with protection from overexposure to UV radiation	The Cancer Council suggested a comprehensive shade policy for all local, government-sponsored, funded or organized community events, and a workplace policy for staff, volunteers and elected officials who participate in outdoor work or activities The SUNbusters initiative provided seeding grants to community and sporting non-profit organizations to build shade for children The Queensland Government Department of Health published technical guidelines for shade provision in public facilities	Established the Ultraviolet Protection Factor (UPF) as a standard for measuring the amount of UVR passing through fabric Manufacturers selling products as sun protective with the UPF label must abide by standards concerning UPF classification UVR passage	UVI and protection times are provided in weather forecasts by the Bureau of Meteorology
New Zealand	Usage banned for minors (<18)	Health and Safety at Work Act 2015 states that employers are required to provide a risk-free environment to prevent injury or illness, including UVR and heat Employers must provide adequate clothing, sunscreen, protective hat and eyewear and water	Employers can claim tax deductions for outdoor sun protection items	Sunscreens must abide by the Australian/New Zealand Sunscreen Standard’s labeling and testing requirements Ingredients and composition must be effective and safe before they are sold	No specific obligations in place The Ministry of Education informs school boards to install sunshades to protect children	Similar to occupational health	The Cancer Society requested that the government fund schools to install shade, and provide them in public spaces, including at events	Established the Ultraviolet Protection Factor (UPF) as a standard to measure the amount of UVR passing through the fabric Manufacturers selling products as sun protective with the UPF label must abide by standards concerning UPF classification UVR passage	A public weather forecasting provides UVI for the day and forecasts
Canada	Usage banned for minors (ages 18–19, depending on province/territory) in all provinces/territories except for Nunavut and Yukon	Limited requirements for heat stress and non-ionizing radiation Some provincial acts provide further specifications	No tax exemptions	Primary Sunscreen Monograph provides the ingredients that could be present and the required labeling such as SPF, cautions and warnings	Most provinces have UVR guidelines within childcare establishments mandated by legislation (protective clothing for outdoor play, application of sunscreen, modeling of sun protective behaviors by staff, provision of shade in outdoor play spaces, scheduling of recess based on sunshine and UV index)	No formal legislation or guidelines The Canadian Dermatology Association (CDA) recommends that sporting activities or training be scheduled outside of 11 AM to 4 PM, that shade be sought, when possible, that sun protective clothing be warned and that sunscreen and lip balm with SPF30+ be applied	11 municipalities have at least one policy that incorporates the provision of natural or artificial shade in land use planning and public facilities The CDA distributed CAD 41,200 in grants to build and install shade structures in outdoor areas	Guidance published by governmental agency indicating that clothing claiming to be UVR-protective should have a UPF rating, with 15 being the lowest	A governmental agency publishes a daily UVI forecast for major cities and towns if the UVI is greater than 1
United States	Strict ban for minors (14–18) in 32 states, parental consent is required in 8 states, 10% tax imposed by the Affordable Care Act of 2010	Occupational Health and Safety provides employers with general duty clauses, e.g., the workplace must be free from recognized hazards that may cause serious harm	6 states have issued tax exemptions for sunscreen products (California, New York, Virginia, Texas, Maryland, and Florida)	Regulated by the FDA Sunscreens containing 16 specified active ingredients are deemed safe and effective	“Shade Planning for America’s Schools” in place to strategize shade planning	The American College of Sports Medicine makes recommendations similar to the CDA	The American Academy of Dermatology issues USD 8000 grants for shade provision projects	Standards address the manufacturing of textile-protective products, their testing, and labeling based on the Australian and New Zealand UPF classification	A governmental agency publishes a daily UVI forecast for major cities and towns
Italy	Usage banned for minors (<18), pregnant women, people with current or previous skin cancer, individuals who do not tan or who burn easily from sun exposure	Only ionizing or artificial radiation are considered to be physical hazards	No tax exemptions	Must have an SPF of at least 6, protect from dangerous UVR, and abide by caution-level labeling requirements	No information available	No information available	No information available	Textiles sold as sun protective must abide by testing and classification EU standards. Products need to have UPF greater than 40 and UVR transmission lower than 5%	Monitoring is carried out by several federal and academic institutions
Germany	Usage banned for minors (<18)	Only ionizing or artificial radiation are considered to be physical hazards	No tax exemptions	Must have an SPF of at least 6, protect from dangerous UVR, and abide by caution-level labeling requirements	“UV protection: Clever in sun and shade” project in place as a source of knowledge and tips for sun protection in children and youth	No information available	No information available	Textiles sold as sun protective must abide by testing and classification EU standards Products need to have UPF greater than 40 and UVR transmission lower than 5%	Monitoring is carried out by several federal institutions
France	Usage banned for minors (<18)	Only ionizing or artificial radiation are considered to be physical hazards	No tax exemptions	Must have an SPF of at least 6, protect from dangerous UVR, and abide by caution-level labeling requirements	No information available	No information available	No information available	Textiles sold as sun protective must abide by testing and classification EU standards Products need to have UPF greater than 40 and UVR transmission lower than 5%	UVI is provided to the public as a forecast instead of measurements, as it is only collected by three stations
United Kingdom	Usage banned for minors (<18)	Only ionizing or artificial radiation are considered to be physical hazards	Tax exemption on MD-prescribed sunscreens	Must have an SPF of at least 6, protect from dangerous UVR, and abide by caution-level labeling requirements	Event Safety Guide asks organizers to consider shade and shelter at dedicated children’s areas Sun Safe Schools national accreditation scheme put in place in 2013 to educate and assist in implementing suitable sun-safe policies Personal, Social, Health and Economic Education curriculum of 2020 made that all English primary schools must educate students about safe and unsafe exposure to the sun and how to reduce the associated risks	No information available	No information available	Textiles sold as sun protective must abide by testing and classification UK standards Products need to have UPF greater than 40	Monitoring carried out by academic institutions on behalf of a governmental department
Japan	No information available	Only ionizing or artificial radiation are considered to be physical hazards	No information available	Regulated by Japanese Law but standards set by the national cosmetic association to measure SP, UVA grade protection, and water resistance are widely followed	No information available	No information available	No information available	Standards in place to test and provide a UPF label for UVR-protective textile	Monitoring is carried out by a governmental agency

**Table 2:** A Summary Table of Legislation Recommendations for Australia, New Zealand and Countries of the G7.

	Commercial Tanning Beds	Occupational Health	Sunscreen Regulation	Early Childhood Services and Schools	Sports and Recreation	Public Shade	UVR-Protective Textiles
Recommendation	Complete ban for all age groups	Minimize risks arising from the work environment through protection policies and practices, training, and the supply of UVR protective equipment at a lower cost (no sales tax)	Remove sales tax on sunscreens, control pricing, and impose labeling and testing requirements to ensure safety and efficacy	Have adequate shaded areas to protect children from overexposure to UVR	Provide individuals with sunscreen and equipment protection from overexposure to UV radiation	Require the presence of shaded areas in all organized outdoor events to protect from overexposure to UV radiation. Such areas can be directly built by the governing entity or funded	Manufacturers selling products as sun protective must have a UPF label that abides by standards concerning UPF classification UVR passage

## References

[1] Conte S., Ghazawi F.M., Le M., Nedjar H., Alakel A., Lagacé F., Mukovozov I.M., Cyr J., Mourad A., Miller W.H. (2022). Population-Based Study Detailing Cutaneous Melanoma Incidence and Mortality Trends in Canada. Front. Med..

[2] Ghazawi F.M., Cyr J., Darwich R., Le M., Rahme E., Moreau L., Netchiporouk E., Zubarev A., Roshdy O., Glassman S.J. (2019). Cutaneous malignant melanoma incidence and mortality trends in Canada: A comprehensive population-based study. J. Am. Acad. Dermatol..

[3] Siegel R.L., Miller K.D., Wagle N.S., Jemal A. (2023). Cancer statistics, 2023. CA Cancer J. Clin..

[4] Forsea A.M. (2020). Melanoma Epidemiology and Early Detection in Europe: Diversity and Disparities. Dermatol. Pract. Concept..

[5] Saginala K., Barsouk A., Aluru J.S., Rawla P., Barsouk A. (2021). Epidemiology of Melanoma. Med. Sci..

[6] Perera E., Gnaneswaran N., Staines C., Win A.K., Sinclair R. (2015). Incidence and prevalence of non-melanoma skin cancer in Australia: A systematic review. Australas. J. Dermatol..

[7] Tang E., Fung K., Chan A.W. (2021). Incidence and mortality rates of keratinocyte carcinoma from 1998–2017: A population-based study of sex differences in Ontario, Canada. CMAJ.

[8] Wu S., Han J., Li W.Q., Li T., Qureshi A.A. (2013). Basal-cell carcinoma incidence and associated risk factors in U.S. women and men. Am. J. Epidemiol..

[9] O’Sullivan D.E., Brenner D.R., Villeneuve P.J., Walter S.D., Demers P.A., Friedenreich C.M., King W.D. (2021). The current burden of non-melanoma skin cancer attributable to ultraviolet radiation and related risk behaviours in Canada. Cancer Causes Control.

[10] Yang D.D., Borsky K., Jani C., Crowley C., Rodrigues J.N., Matin R.N., Marshall D.C., Salciccioli J.D., Shalhoub J., Goodall R. (2023). Trends in keratinocyte skin cancer incidence, mortality and burden of disease in 33 countries between 1990 and 2017. Br. J. Dermatol..

[11] Global Cancer Observatory, International Agency for Research on Cancer, World Health Organization Estimated Number of New Cases in 2020, Melanoma of Skin, Non-Melanoma Skin Cancer, Both Sexes, All Ages. https://gco.iarc.fr/today/online-analysis-table?v=2020&mode=population&mode_population=countries&population=900&populations=900&key=asr&sex=0&cancer=16_17&type=0&statistic=5&prevalence=0&population_group=0&ages_group%5B%5D=0&ages_group%5B%5D=17&group_cancer=1&include_nmsc=1&include_nmsc_other=1.

[12] Marks R. (1990). Skin cancer control in the 1990’s, from slip! Slop! Slap! To sun smart. Australas. J. Dermatol..

[13] Blazek K., Furestad E., Ryan D., Damian D., Fernandez-Penas P., Tong S. (2022). The impact of skin cancer prevention efforts in New South Wales, Australia: Generational trends in melanoma incidence and mortality. Cancer Epidemiol..

[14] Tabbakh T., Volkov A., Wakefield M., Dobbinson S. (2019). Implementation of the SunSmart program and population sun protection behaviour in Melbourne, Australia: Results from cross-sectional summer surveys from 1987 to 2017. PLoS Med..

[15] Australian Institute of Health and Welfare (2016). Skin Cancer in Australia.

[16] Olsen C.M., Pandeya N., Green A.C., Ragaini B.S., Venn A.J., Whiteman D.C. (2022). Keratinocyte cancer incidence in Australia: A review of population-based incidence trends and estimates of lifetime risk. Public Health Res. Pract..

[17] Burris S., Wagenaar A.C., Swanson J., Ibrahim J.K., Wood J., Mello M.M. (2010). Making the Case for Laws That Improve Health: A Framework for Public Health Law Research. Milbank Q..

[18] Gostin L.O. (2000). Public Health Law in a New CenturyPart I: Law as a Tool to Advance the Community’s Health. JAMA.

[19] Burris S., Ashe M., Levin D., Penn M., Larkin M. (2016). A Transdisciplinary Approach to Public Health Law: The Emerging Practice of Legal Epidemiology. Annu. Rev. Public Health.

[20] Diehl K., Lindwedel K.S., Mathes S., Görig T., Gefeller O. (2022). Tanning Bed Legislation for Minors: A Comprehensive International Comparison. Children.

[21] Hartsfield D., Moulton A.D., McKie K.L. (2007). A review of model public health laws. Am. J. Public Health.

[22] Dessinioti C., Stratigos A.J. (2022). An Epidemiological Update on Indoor Tanning and the Risk of Skin Cancers. Curr. Oncol..

[23] Le Clair M.Z., Cockburn M.G. (2016). Tanning bed use and melanoma: Establishing risk and improving prevention interventions. Prev. Med. Rep..

[24] Cancer Council Australia Position Statement—Private Ownership and Use of Solariums in Australia. https://wiki.cancer.org.au/policy/Position_statement_-_Private_solariums.

[25] Government of Canada Tanning Beds and Equipment. https://www.canada.ca/en/health-canada/services/sun-safety/tanning-beds-lamps.html.

[26] Internal Revenue Service Excise Tax on Indoor Tanning Services. https://www.irs.gov/businesses/small-businesses-self-employed/excise-tax-on-indoor-tanning-services-frequently-asked-questions.

[27] Stanganelli I., Gandini S., Magi S., Mazzoni L., Medri M., Agnoletti V., Lombi L., Falcini F. (2013). Sunbed use among subjects at high risk of melanoma: An Italian survey after the ban. Br. J. Dermatol..

[28] (1956). Health Act 1956.

[29] Wikipedia Ganguro. https://en.wikipedia.org/wiki/Ganguro#:~:text=Ganguro%20(Japanese%3A%20ガングロ)%20is,up%20liberally%20applied%20by%20fashionistas.

[30] Eden M., Hainsworth R., Gordon L.G., Epton T., Lorigan P., Rhodes L.E., Marais R., Green A.C., Payne K. (2022). Cost-effectiveness of a policy-based intervention to reduce melanoma and other skin cancers associated with indoor tanning. Br. J. Dermatol..

[31] Eskander A., Marqueen K.E., Edwards H.A., Joshua A.M., Petrella T.M., de Almeida J.R., Goldstein D.P., Ferket B.S. (2021). To ban or not to ban tanning bed use for minors: A cost-effectiveness analysis from multiple US perspectives for invasive melanoma. Cancer.

[32] Gordon L.G., Sinclair C., Cleaves N., Makin J.K., Rodriguez-Acevedo A.J., Green A.C. (2020). Consequences of banning commercial solaria in 2016 in Australia. Health Policy.

[33] Worksafe New Zealand Protecting Workers from Solar UV Radiation. https://www.worksafe.govt.nz/topic-and-industry/work-related-health/carcinogens-and-airborne-risks/protecting-workers-from-solar-uv-radiation/.

[34] Worksafe New Zealand Introduction to the Health and Safety at Work Act 2015—Special Guide. https://www.worksafe.govt.nz/managing-health-and-safety/getting-started/introduction-hswa-special-guide/#lf-doc-23779.

[35] John S.M., Garbe C., French L.E., Takala J., Yared W., Cardone A., Gehring R., Spahn A., Stratigos A. (2021). Improved protection of outdoor workers from solar ultraviolet radiation: Position statement. J. Eur. Acad. Dermatol. Venereol..

[36] Bauer A., Diepgen T.L., Schmitt J. (2011). Is occupational solar ultraviolet irradiation a relevant risk factor for basal cell carcinoma? A systematic review and meta-analysis of the epidemiological literature. Br. J. Dermatol..

[37] Schmitt J., Seidler A., Diepgen T.L., Bauer A. (2011). Occupational ultraviolet light exposure increases the risk for the development of cutaneous squamous cell carcinoma: A systematic review and meta-analysis. Br. J. Dermatol..

[38] International Commission on Non-Ionizing Radiation Protection (2007). Protecting Workers from Ultraviolet Radiation.

[39] Alfonso J.H., Martinsen J.I., Weiderpass E., Pukkala E., Kjaerheim K., Tryggvadottir L., Lynge E. (2021). Occupation and cutaneous melanoma: A 45-year historical cohort study of 14·9 million people in five Nordic countries. Br. J. Dermatol..

[40] Maduka R.C., Tai K., Gonsai R., DeWalt N., Chetty A., Brackett A., Olino K., Schneider E.B., Ahuja V. (2023). Indoor Versus Outdoor: Does Occupational Sunlight Exposure Increase Melanoma Risk? A Systematic Review. J. Surg. Res..

[41] Gambichler T., Altmeyer P., Hoffmann K. (2002). Role of clothes in sun protection. Recent Results Cancer Res..

[42] Harrison S.L., Downs N. (2015). Development of a Reproducible Rating System for Sun Protective Clothing That Incorporates Body Surface Coverage. World J. Eng. Technol..

[43] Roy C.R., Gies P.H., McLennan A. (2002). Sun protective clothing: 5 years of experience in Australia. Recent Results Cancer Res..

[44] Australian Radiation Protection and Nuclear Safety Agency New Zealand Sun Protective Clothing. https://www.arpansa.gov.au/our-services/testing-and-calibration/ultraviolet-services/labelling-sun-protective-clothing/nz-standard.

[45] Australian Radiation Protection and Nuclear Safety Agency Australian Sun Protective Clothing. https://www.arpansa.gov.au/our-services/testing-and-calibration/ultraviolet-services/labelling-sun-protective-clothing/au-standard.

[46] Downs N.J., Harrison S.L. (2018). A comprehensive approach to evaluating and classifying sun-protective clothing. Br. J. Dermatol..

[47] Eurofins Consumer Clothing with a UV Protection Claim: PPE Regulations or Not. https://www.eurofins.com/consumer-product-testing/media-centre/news/consumer-clothing-with-a-uv-protection-claim-ppe-regulations-or-not/.

[48] England P.H. Ultraviolet Radiation: Frequently Asked Questions. https://www.gov.uk/government/publications/ultraviolet-radiation-and-sunscreen/ultraviolet-radiation-frequently-asked-questions.

[49] Hatch K.L. (2002). American standards for UV-protective textiles. Recent Results Cancer Res..

[50] Government of Canada (2007). UV Protective Clothing: Educate Yourself before Buying.

[51] GlobalSpec Textiles—Evaluation Method of Ultraviolet Ray-Shielding. https://standards.globalspec.com/std/13351063/jis-l-1925.

[52] Australian Government, Department of Health and Aged Care Sunscreen Regulation in Australia. https://www.tga.gov.au/sunscreen-regulation-australia.

[53] Australian Radiation Protection and Nuclear Safety Agency Sun Protection Using Sunscreens. https://www.arpansa.gov.au/understanding-radiation/radiation-sources/more-radiation-sources/sun-protection-sunscreen.

[54] Muller T. (2021). Sunscreen (Product Safety Standard) Bill. https://www.legislation.govt.nz/bill/member/2021/0011/latest/whole.html.

[55] U.S. Food and Drug Administration An Update on Sunscreen Requirements: The Deemed Final Order and the Proposed Order. https://www.fda.gov/drugs/news-events-human-drugs/update-sunscreen-requirements-deemed-final-order-and-proposed-order.

[56] Health Canada Primary Sunscreen Monograph. https://webprod.hc-sc.gc.ca/nhpid-bdipsn/atReq.do?atid=sunscreen-ecransolaire.

[57] European Commission Sunscreen Products. https://single-market-economy.ec.europa.eu/sectors/cosmetics/cosmetic-products-specific-topics/sunscreen-products_en.

[58] Office for Product Safety and Standards Regulation 2009/1223 and the Cosmetic Products Enforcement Regulations 2013: Great Britain. https://www.gov.uk/government/publications/cosmetic-products-enforcement-regulations-2013/regulation-20091223-and-the-cosmetic-products-enforcement-regulations-2013-great-britain.

[59] Nakai K., Tanaka T. (2015). Introducing Internet Retailing of OTC Drugs in Japan: Revision of the Pharmaceutical Affairs Law. Ther. Innov. Regul. Sci..

[60] Japan Cosmetic Industry Association Technical Committee JCIA Standards and Related Notifications for Sun Protection Factor (SPF), UVA Protection Grade (PA) and Water Resistance for UV Protection. https://www.jcia.org/en/common/download/top/jcia-notifications-spf-pa.pdf.

[61] Weig E.A., Tull R., Chung J., Brown-Joel Z.O., Majee R., Ferguson N.N. (2020). Assessing factors affecting sunscreen use and barriers to compliance: A cross-sectional survey-based study. J. Dermatol. Treat..

[62] Mahé E., Beauchet A., de Maleissye M.F., Saiag P. (2011). Are sunscreens luxury products?. J. Am. Acad. Dermatol..

[63] Walker H., Maitland C., Tabbakh T., Preston P., Wakefield M., Sinclair C. (2022). Forty years of Slip! Slop! Slap! A call to action on skin cancer prevention for Australia. Public Health Res. Pract..

[64] McNoe B.M., Gage R., Signal L. (2022). What can Aotearoa New Zealand learn from the Australian Sunsmart Story? A qualitative study. Aust. N. Z. J. Public Health.

[65] Manitoba Government (2013). Information Bulletin: The Retail Sales Tax Act.

[66] California Department of Tax and Fee Administration Laws, Regulations and Annotations. https://www.cdtfa.ca.gov/lawguides/vol2/suta/425-0823-5.html.

[67] New York State Department of Taxation and Finance Drugstores and Pharmacies: Tax Bulletin ST-193. https://www.tax.ny.gov/pubs_and_bulls/tg_bulletins/st/drugstores.htm.

[68] Texas Comptroller of Public Accounts Sales Tax Exemptions for Over-the-Counter Drugs and Medicines: Publication # 94-155. https://star.comptroller.texas.gov/view/201211676L.

[69] Virginia Department of Taxation Tax Bulletin 13-5: Information Regarding Nonprescription Drugs and Proprietary Medicines. https://www.tax.virginia.gov/laws-rules-decisions/tax-bulletins/13-5.

[70] The Comptroller of Maryland (2023). List of Tangible Personal Property and Services Subject to Sales and Use Tax.

[71] Florida Department of Revenue Freedom Week: 2022 Sales Tax Holiday. https://floridarevenue.com/FreedomWeek/Pages/default.aspx.

[72] New Jersey State New Jersey Sales Tax Guide. https://www.state.nj.us/treasury/taxation/pdf/pubs/sales/su4.pdf.

[73] Vermont Government FS-1028—General Guidelines on Sales Tax: What Is Taxable and Exempt. https://tax.vermont.gov/content/fs-1028-general-guidelines-sales-tax-what-taxable-and-exempt.

[74] Chartered Institute of Taxation Sunscreen Won’t Be Made VAT-Exempt, Says Financial Secretary, Despite MP’s Campaign. https://www.tax.org.uk/sunscreen-wont-be-made-vat-exempt.

[75] Knaul F., Nugent R. (2006). Fiscal Policies for Health Promotion and Disease Prevention. Disease Control Priorities in Developing Countries.

[76] Gelius P., Messing S., Tcymbal A., Whiting S., Breda J., Abu-Omar K. (2021). Policy Instruments for Health Promotion: A Comparison of WHO Policy Guidance for Tobacco, Alcohol, Nutrition and Physical Activity. Int. J. Health Policy Manag..

[77] Wright A., Smith K.E., Hellowell M. (2017). Policy lessons from health taxes: A systematic review of empirical studies. BMC Public Health.

[78] Baum F., Fisher M. (2014). Why behavioural health promotion endures despite its failure to reduce health inequities. Sociol. Health Illn..

[79] Early Childhood on Stafford Sun Protection Policy. https://earlychildhoodonstafford.co.nz/sun-protection-policy/.

[80] New Zealand Government (2008). Education (Early Childhood Services) Regulations.

[81] Te Tahuhu o te Matauranga Shade and Shelter at Schools. https://www.education.govt.nz/school/property-and-transport/projects-and-design/design/general-design-guidance/shade-structures/.

[82] Gage R., Leung W., Stanley J., Reeder A., Mackay C., Smith M., Barr M., Chambers T., Signal L. (2018). Sun Protection among New Zealand Primary School Children. Health Educ. Behav..

[83] McNoe B.M., Reeder A.I. (2019). Sun protection policies and practices in New Zealand primary schools. N. Z. Med. J..

[84] Seidel N., Fieber V., Breitbart E.W., Bornhäuser M., Stölzel F. (2021). Cluster Randomized Trial: Sun Protection Intervention ‘Clever in Sun and Shade for Preschools’-Effectiveness and Dissemination. Children.

[85] Baig I.T., Petronzio A., Maphet B., Chon S. (2023). A Review of the Impact of Sun Safety Interventions in Children. Dermatol. Pract. Concept..

[86] Heckman C.J., Liang K., Riley M. (2019). Awareness, understanding, use, and impact of the UV index: A systematic review of over two decades of international research. Prev. Med..

[87] Fioletov V., Kerr J.B., Fergusson A. (2010). The UV index: Definition, distribution and factors affecting it. Can. J. Public Health.

[88] Kanellis V.G. (2019). Ultraviolet radiation sensors: A review. Biophys. Rev..

[89] Australian Government Bureau of Meteorology About UV and Sun Protection Times. http://www.bom.gov.au/uv/.

[90] NIWA Taihoro Nukurangi Today’s UV Index. https://niwa.co.nz/our-services/online-services/uv-and-ozone/todays-uv-index.

[91] Government of Canada How to Find Your UV Index. https://www.canada.ca/en/environment-climate-change/services/weather-health/uv-index-sun-safety/how-to-find.html.

[92] United States Environmental Protection Agency UV Index. https://www.epa.gov/sunsafety/uv-index-1.

[93] Schmalwieser A.W., Gröbner J., Blumthaler M., Klotz B., De Backer H., Bolsée D., Werner R., Tomsic D., Metelka L., Eriksen P. (2017). UV Index monitoring in Europe. Photochem. Photobiol. Sci..

[94] Japan Meteorological Agency UV Index. https://www.data.jma.go.jp/gmd/env/uvindex/en/index.html?elem=0&area=0&lat=38.42777351132902&lng=141.77856445312503&zoom=6.

[95] Morton S.K., Harrison S.L. (2022). Slip, Slop, Slap, Slide, Seek and Sport: A Systematic Scoping Review of Sun Protection in Sport in Australasia. Curr. Oncol..

[96] Can Too You Can Too Protect Yourself This Summer. https://www.cantoo.org.au/blog/you-can-too-protect-yourself-this-summer.

[97] Gage R., Barr M., Stanley J., Reeder A., Mackay C., Smith M., Chambers T., Leung W., Signal L. (2018). Sun protection and shade availability in New Zealand’s outdoor recreation spaces. N. Z. Med. J..

[98] Lewis O. Skin Cancer Group Rolls Out Free Public Sunscreen Dispensers in Christchurch. https://www.stuff.co.nz/national/health/108066525/skin-cancer-group-rolls-out-free-public-sunscreen-dispensers-in-christchurch?fbclid=IwAR1ftJ6ua2lUbn1fo1y5QY5VgP8lpPxrC8EkCOV-XIclTKtxsLCmZ3KDr8k.

[99] Save Your Skin Foundation 2022 Sunscreen Dispenser Pilot. https://saveyourskin.ca/tag/skin-cancer-prevention/.

[100] Los Angeles County Beaches & Harbors Annual Beach Parking Pass. https://beaches.lacounty.gov/annual-beach-parking-pass/.

[101] Quinn M. Forget the Sunscreen? It’s Free Now in Some Cities. https://www.governing.com/archive/gov-sunscreen-dispensers-miami-boston.html.

[102] Eason C.D., Rundle C., Dunnick C.A., Hugh J., Dellavalle R.P. (2021). National trends in free public sunscreen dispensers. J. Am. Acad. Dermatol..

[103] Rosso S., Zanetti R., Martinez C., Tormo M.J., Schraub S., Sancho-Garnier H., Franceschi S., Gafà L., Perea E., Navarro C. (1996). The multicentre south European study ‘Helios’. II: Different sun exposure patterns in the aetiology of basal cell and squamous cell carcinomas of the skin. Br. J. Cancer.

[104] Rosso S., Joris F., Zanetti R. (1999). Risk of basal and squamous cell carcinomas of the skin in Sion, Switzerland: A case-control study. Tumori J..

[105] Kaskel P., Lange U., Sander S., Huber M.A., Utikal J., Leiter U., Krähn G., Meurer M., Kron M. (2015). Ultraviolet exposure and risk of melanoma and basal cell carcinoma in Ulm and Dresden, Germany. J. Eur. Acad. Dermatol. Venereol..

[106] Loria D., Matos E. (2001). Risk factors for cutaneous melanoma: A case-control study in Argentina. Int. J. Dermatol..

[107] Collins L., Earl C., Stewart D., Stoneham M. (2001). Preventing Skin Cancer in Queensland: An Evaluation of a Community Shade Creation Project. Environ. Health.

[108] Cancer Council Queensland SunSmart Shade Creation Initiative. https://cancerqld.org.au/cancer-prevention/programs-resources/shade-initiative/.

[109] Victoria Department of Health Shade Grants Program. https://www.health.vic.gov.au/preventive-health/shade-grants-program.

[110] Queensland Government Technical Guidelines for Shade Provision in Public Facilities. https://www.health.qld.gov.au/__data/assets/pdf_file/0024/443931/tech-guidelines-shade-provision.pdf.

[111] Cancer Society Shade in Public Places. https://www.cancer.org.nz/about-us/our-advocacy-work/our-advocacy-campaigns/shade-in-public-places/.

[112] Gage R., O’Toole C., Robinson A., Reeder A., Signal L., Mackay C. (2018). Wellington Playgrounds Uncovered: An Examination of Solar Ultraviolet Radiation and Shade Protection in New Zealand. Photochem. Photobiol..

[113] American Academy of Dermatology Association Shade Structure Grant Program. Aad.org/public-health/shade-structure-grants.

[114] Holman D.M., Kapelos G.T., Shoemaker M., Watson M. (2018). Shade as an Environmental Design Tool for Skin Cancer Prevention. Am. J. Public Health.

[115] McKenzie C., Nahm W.J., Kearney C.A., Zampella J.G. (2023). Sun-protective behaviors and sunburn among US adults. Arch. Dermatol. Res..

[116] Irmak C., Murdock M.R., Kanuri V.K. (2020). When Consumption Regulations Backfire: The Role of Political Ideology. J. Mark. Res..

[117] Kitching J., Hart M., Wilson N. (2015). Burden or benefit? Regulation as a dynamic influence on small business performance. Int. Small Bus. J..

[118] Guasch J.L., Hahn R.W. (1999). The Costs and Benefits of Regulation: Implications for Developing Countries. World Bank Res. Obs..

[119] Holick M.F. (2020). Sunlight, UV Radiation, Vitamin D, and Skin Cancer: How Much Sunlight Do We Need?. Adv. Exp. Med. Biol..

[120] Mead M.N. (2008). Benefits of sunlight: A bright spot for human health. Environ. Health Perspect..

[121] Morrison R. (2021). Sunbathing Makes You Horny! Exposure to Sunlight Enhances Romantic Passion by Boosting the Release of Sexual Hormones, Study Finds. https://www.dailymail.co.uk/sciencetech/article-9926477/Exposure-sunlight-enhances-romantic-passion-boosting-release-sexual-hormones.html.

[122] Parikh R., Sorek E., Parikh S., Michael K., Bikovski L., Tshori S., Shefer G., Mingelgreen S., Zornitzki T., Knobler H. (2021). Skin exposure to UVB light induces a skin-brain-gonad axis and sexual behavior. Cell. Rep..

[123] Luo C.W., Chen S.P., Chiang C.Y., Wu W.J., Chen C.J., Chen W.Y., Kuan Y.H. (2022). Association between Ultraviolet B Exposure Levels and Depression in Taiwanese Adults: A Nested Case-Control Study. Int. J. Environ. Res. Public Health.

[124] Toledo A., Karppinen T., Miettinen M.E., Leppäluoto J., Vuolteenaho O., Ylianttila L., Kautiainen H., Snellman E., Partonen T. (2019). Narrow-band ultraviolet B (NB UV-B) exposures improve mood in healthy individuals differently depending on chronotype. Chronobiol. Int..

[125] Veleva B.I., van Bezooijen R.L., Chel V.G.M., Numans M.E., Caljouw M.A.A. (2018). Effect of ultraviolet light on mood, depressive disorders and well-being. Photodermatol. Photoimmunol. Photomed..

[126] Fell G.L., Robinson K.C., Mao J., Woolf C.J., Fisher D.E. (2014). Skin β-endorphin mediates addiction to UV light. Cell.

[127] Robinson K.C., Fisher D.E. (2014). Tanning as a substance abuse. Commun. Integr. Biol..

[128] Canadian Cancer Society Survival Statistics for Non-Melanoma Skin Cancer. https://cancer.ca/en/cancer-information/cancer-types/skin-non-melanoma/prognosis-and-survival/survival-statistics#:~:text=Relative%20survival&text=Survival%20for%20most%20non%2Dmelanoma,survival%20for%20BCC%20is%20100%25.

[129] Canadian Cancer Society Survival Statistics for Melanoma Skin Cancer. https://cancer.ca/en/cancer-information/cancer-types/skin-melanoma/prognosis-and-survival/survival-statistics.

[130] Canadian Cancer Society Survival Statistics for Colorectal Cancer. https://cancer.ca/en/cancer-information/cancer-types/colorectal/prognosis-and-survival/survival-statistics.

[131] Canadian Cancer Society Survival Statistics for Breast Cancer. https://cancer.ca/en/cancer-information/cancer-types/breast/prognosis-and-survival/survival-statistics.

[132] Canadian Cancer Society Survival Statistics for Ovarian Cancer. https://cancer.ca/en/cancer-information/cancer-types/ovarian/prognosis-and-survival/survival-statistics.

[133] Peixoto R.D., Oliveira L.J.C., Passarini T.M., Andrade A.C., Diniz P.H., Prolla G., Amorim L.C., Gil M., Lino F., Garicochea B. (2022). Vitamin D and colorectal cancer—A practical review of the literature. Cancer Treat. Res. Commun..

[134] Kao S.Z., Ekwueme D.U., Holman D.M., Rim S.H., Thomas C.C., Saraiya M. (2023). Economic burden of skin cancer treatment in the USA: An analysis of the Medical Expenditure Panel Survey Data, 2012–2018. Cancer Causes Control.

[135] Savoye I., Olsen C.M., Whiteman D.C., Bijon A., Wald L., Dartois L., Clavel-Chapelon F., Boutron-Ruault M.C., Kvaskoff M. (2018). Patterns of Ultraviolet Radiation Exposure and Skin Cancer Risk: The E3N-SunExp Study. J. Epidemiol..

[136] Lerche C.M., Togsverd-Bo K., Philipsen P.A., Wulf H.C. (2017). Impact of UVR Exposure Pattern on Squamous Cell Carcinoma-A Dose-Delivery and Dose-Response Study in Pigmented Hairless Mice. Int. J. Mol. Sci..

[137] Qureshi A.A., Laden F., Colditz G.A., Hunter D.J. (2008). Geographic variation and risk of skin cancer in US women. Differences between melanoma, squamous cell carcinoma, and basal cell carcinoma. Arch. Intern. Med..

[138] Cai H., Sobue T., Kitamura T., Sawada N., Iwasaki M., Shimazu T., Tsugane S. (2020). Epidemiology of nonmelanoma skin cancer in Japan: Occupational type, lifestyle, and family history of cancer. Cancer Sci..

[139] Håkansson N., Floderus B., Gustavsson P., Feychting M., Hallin N. (2001). Occupational sunlight exposure and cancer incidence among Swedish construction workers. Epidemiology.

[140] Kenborg L., Jørgensen A.D., Budtz-Jørgensen E., Knudsen L.E., Hansen J. (2010). Occupational exposure to the sun and risk of skin and lip cancer among male wage earners in Denmark: A population-based case-control study. Cancer Causes Control.

[141] Ezer T., Patel P. (2018). Strategic Litigation to Advance Public Health. Health Hum. Rights.

